# Induction of SOCS3 by liver X receptor suppresses the proliferation of hepatocellular carcinoma cells

**DOI:** 10.18632/oncotarget.19321

**Published:** 2017-07-18

**Authors:** Haojun Xiong, Yan Zhang, Shan Chen, Zhenhong Ni, Jintao He, Xinzhe Li, Bo Li, Kai Zhao, Fan Yang, Yijun Zeng, Bingbo Chen, Fengtian He

**Affiliations:** ^1^ Department of Biochemistry and Molecular Biology, College of Basic Medical Sciences, Third Military Medical University, Chongqing 400038, China; ^2^ Battalion 17 of Students, College of Preventive Medicine, Third Military Medical University, Chongqing 400038, China; ^3^ Laboratory Animal Center, Third Military Medical University, Chongqing 400038, China

**Keywords:** HCC, LXR, SOCS3

## Abstract

Liver X receptor (LXR), a member of nuclear receptor superfamily, is involved in the regulation of glucose, lipid and cholesterol metabolism. Recently, it has been reported that LXR suppress different kinds of cancers including hepatocellular carcinoma (HCC). However, the corresponding mechanism is still not well elucidated. In the present study, we found that activation of LXR downregulated cyclin D1 while upregulated p21 and p27 by elevating the level of suppressor of cytokine signaling 3 (SOCS3), leading to the cell cycle arrest at G1/S phase and growth inhibition of HCC cells. Moreover, we demonstrated that LXRα (not LXRβ) mediated the induction of SOCS3 in HCC cells. Subsequently, we showed that LXR activation enhanced the mRNA stability of SOCS3, but had no significant influence on the transcriptional activity of *SOCS3* gene promoter. The experiments in nude mice revealed that LXR agonist inhibited the growth of xenograft tumors and enhanced SOCS3 expression *in vivo*. These results indicate that “LXRα-SOCS3-cyclin D1/p21/p27” is a novel pathway by which LXR exerts its anti-HCC effects, suggesting that the pathway may be a new potential therapeutic target for HCC treatment.

## INTRODUCTION

Liver X receptor (LXR) belongs to a subgroup of ligand-activated nuclear receptor superfamily [1]. LXR contain two isoforms, LXRα (also known as NR1H3) and LXRβ (also known as NR1H2) [2]. The expression profile of LXR isoforms in human organs are quite different, LXRα is highly expressed in the liver, intestine, kidney and adipose tissues whereas LXRβ is ubiquitously expressed in almost all tissues at a low concentration [3]. In most circumstances, LXR binds with retinoic X receptor (RXR) to form heterodimer after activation, and then the heterodimer LXR/RXR binds to specific DNA sequences called LXR response elements (LXRE) and regulates gene expression [3]. Oxysterols, mono-oxygenated derivatives of cholesterol, are a potential endogenous agonists for LXR, whereas TO90137 and GW3965 are exogenous synthetic agonists commonly used in experimental studies. Previous reports have shown that LXR plays an important role in the regulation of lipid metabolism as it can promote the expression of key genes (*SREBP-1c*, *ChREBP*, *FASN* et al) critical for hepatic lipogenesis [4]. In addition, LXR activation can reduce the body load of cholesterol through upregulating important genes (*ABCA1*, *ABCG1*, *ABCG5* and *ABCG8*) involved in reverse cholesterol transport [5]. Interestingly, recent studies have demonstrated that LXR has an anti-neoplastic function in various human cancers such as prostate cancer [6], breast cancer [7], ovarian carcinoma [8], skin cancer [9], colon cancer [10], and HCC [11]. However, the molecular mechanisms by which LXR exerts its anti-cancer effects are still not that clear, which needs to be explored urgently.

SOCS3 is one of the SOCS family members containing eight proteins (SOCS1-7 and CISH) [12]. All members of SOCS family share the same structure, a variable N-terminal region, a central Src homology 2 (SH2) domain and a conserved C-terminal region called SOCS box. SOCS proteins can suppress the JAK/STAT signaling pathway through the following three mechanisms: (1) SOCS1 and SOCS3 inhibit the activity of JAKs through kinase inhibitory region (KIR) which acts as a pseudo-substrate [13]. (2) SOCS proteins can compete for the receptor (JAKs) motifs that are essential for the phosphorylation of STATs through SH2 domain [14]. (3) SOCS proteins can mediate the ubiquitination of their substrates through the SOCS box, leading to the degradation of the substrates via proteasome-dependent pathway [15]. Among the SOCS family members, SOCS3 and SOCS1 are the most extensively studied. Previous reports have shown that SOCS3 regulates numerous biological and pathological processes such as inflammation [16], immunity [17], diabetes [18], angiogenesis [19] and development [20]. Recently, more and more studies focus on the anti-cancer effect of SOCS3 [21]. It has been reported that SOCS3 has the potential of anti-tumor in many human cancers such as breast cancer [22], ovarian cancer [23] and HCC [24]. However, it is still unknown whether the anti-HCC effect of SOCS3 could be regulated by LXR.

In this study, we demonstrated that activation of LXR induced the expression of SOCS3, leading to the downregulation of cyclin D1 and upregulation of p21 and p27, which contributed to the cell cycle arrest at G1/S phase and growth inhibition of HCC cells. Moreover, we found that LXR agonist inhibited the growth of HCC xenografts and enhanced SOCS3 expression *in vivo*. These results indicate that “LXRα-SOCS3-cyclin D1/p21/p27” is a novel pathway by which LXR exerts its anti-HCC effects, suggesting that the pathway may be a new potential therapeutic target for HCC treatment.

## RESULTS

### The expression of SOCS3 is downregulated in HCC tissues and cells

**Figure 1:**
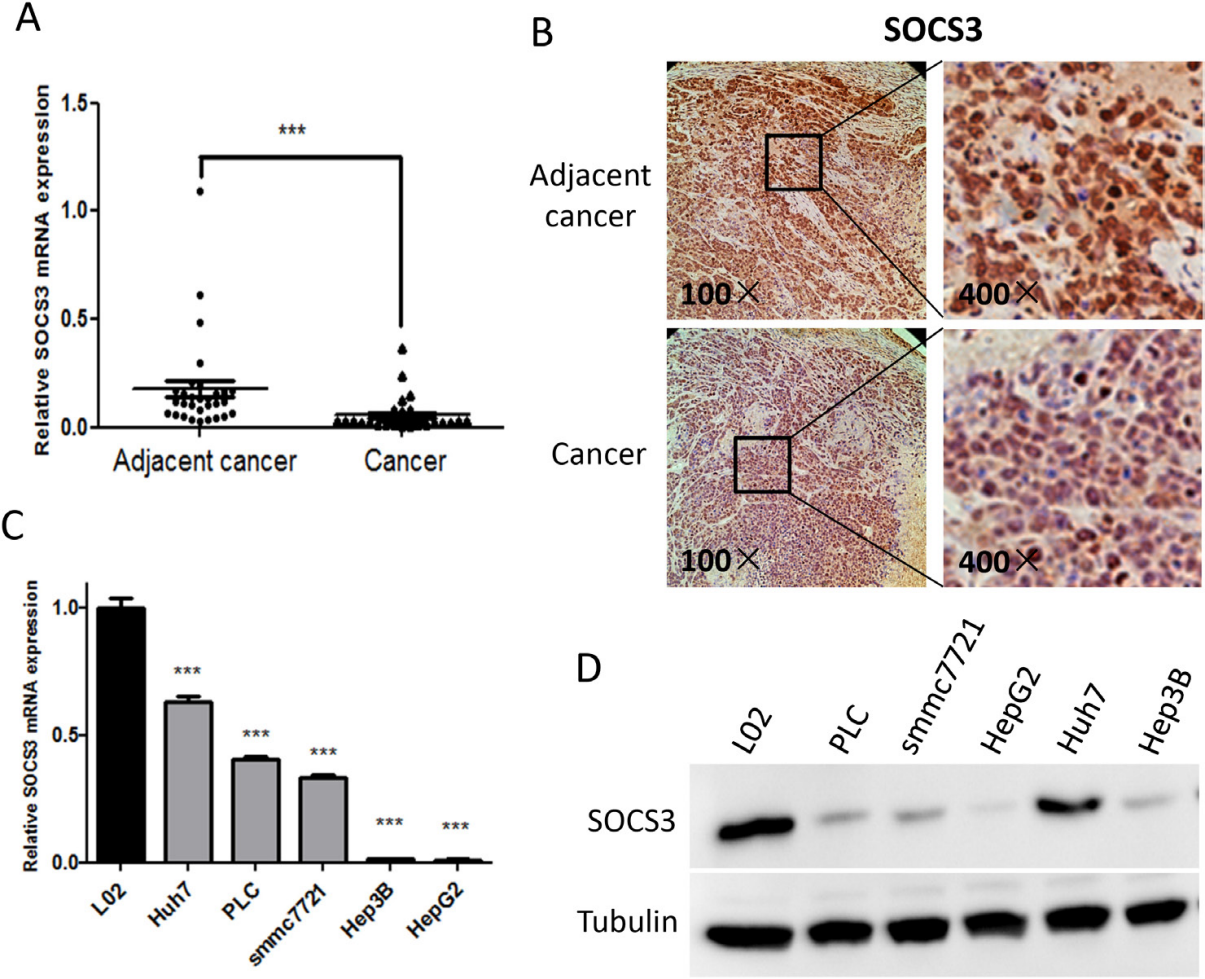
The expression of SOCS3 is down-regulated in HCC tissues and cells **(A)** qPCR analysis of SOCS3 mRNA in 30 HCC tissues and the corresponding adjacent normal tissues, taking β-actin mRNA as an endogenous control. **(B)** Immunohistochemistry analysis of SOCS3 protein in formalin-fixed paraffin-embedded clinical HCC tissues and the corresponding adjacent normal tissues, and the representative images were shown. **(C, D)** The expression profiles of SOCS3 mRNA and protein were separately examined by qPCR **(C)** and Western blot **(D)** in 5 HCC cell lines and relatively normal hepatic cell line L02. ***P < 0.001.

To determine the expression profile of SOCS3 in HCC tissues, 30-paired HCC and the corresponding adjacent normal tissues were utilized. As shown in Figure 1A, the mRNA level of SOCS3 was remarkably decreased in HCC tissues compared to the corresponding non-tumorous tissues. Immunohistochemistry (IHC) revealed that the level of SOCS3 protein was also dramatically downregulated in HCC tissues (Figure 1B). Moreover, we found that the levels of SOCS3 mRNA (Figure 1C) and protein (Figure 1D) were notably decreased in five HCC cell lines (HepG2, Hep3B, Huh7, PLC and smmc7721) compared to the relatively normal cell line L02. These results indicated that the expression of SOCS3 was significantly downregulated in HCC tissues and cells.

### LXR agonists elevate the mRNA level of SOCS3 in HCC cells

**Figure 2:**
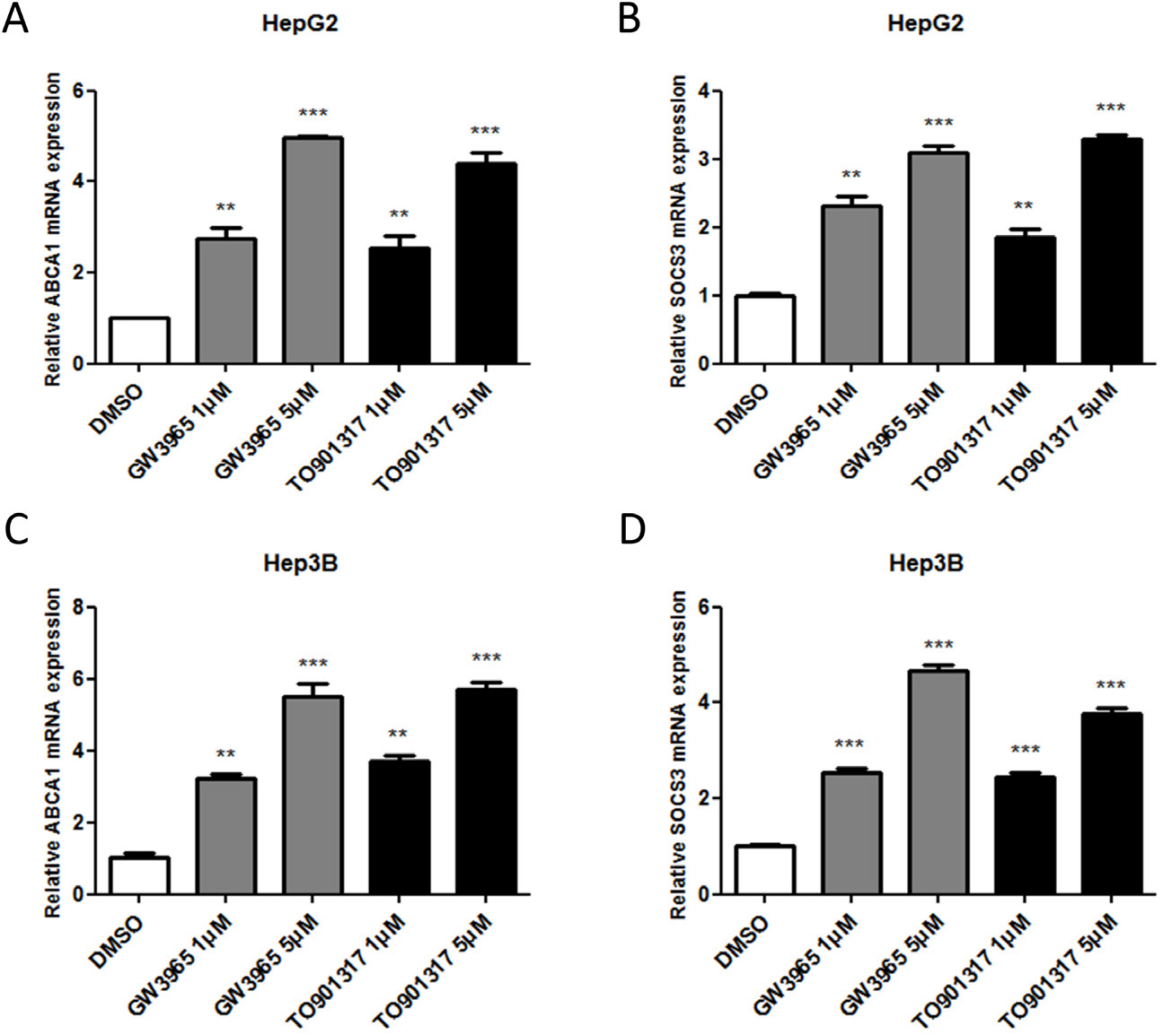
LXR agonists elevate the mRNA level of SOCS3 in HCC cells HepG2 and Hep3B cells were cultured in complete medium for 12h and then treated with DMSO (control) or different concentrations (1μM and 5μM) of LXR agonists (GW3965 and TO901317) for 24h. Then the mRNA levels of ABCA1 **(A, C)** and SOCS3 **(B, D)** cells were determined by qPCR, taking β-actin mRNA as an endogenous control. **P < 0.01; ***P < 0.001.

To investigate whether LXR was involved in the induction of SOCS3 mRNA in HCC cells, quantitative PCR (qPCR) was performed after treatment with LXR agonists in HepG2 and Hep3B cells. ABCA1, a well-known target gene of LXR [25], was taken as a positive control in the experiments. As shown Figure 2, LXR agonists (GW3965 and TO901317) markedly increased the mRNA levels of ABCA1 (Figure 2A and 2C) and SOCS3 (Figure 2B and 2D) in a dose-dependent manner in both HepG2 cells (Figure 2A and 2B) and Hep3B cells (Figure 2C and 2D). These results proved that LXR agonists elevated the mRNA level of SOCS3 in HCC cells.

### LXR agonists upregulate SOCS3 protein via activating LXRα

**Figure 3:**
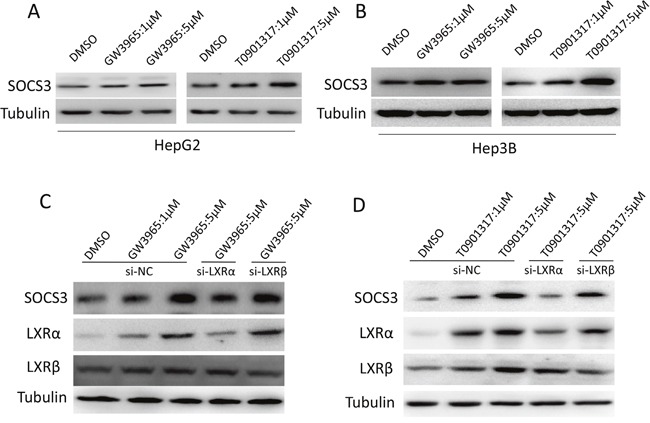
LXR agonists upregulate SOCS3 protein via activating LXRα **(A, B)** After cultured in complete medium for 12 h, HepG2 **(A)** and Hep3B **(B)** cells were treated with DMSO or the indicated concentrations of LXR agonists (GW3965 and TO901317) for 24 h. Then the level of SOCS3 protein were assayed by Western blot. **(C, D)** After transiently transfected with the siRNA for LXRα or LXRβ (si-LXRα or si-LXRβ) or negative control siRNA (si-NC) for 12 h, HepG2 cells were treated with DMSO or the indicated concentrations of GW3965 **(C)** or TO901317 **(D)** for 24 h. Then the level of SOCS3 protein was determined by Western blot.

To clarify whether the protein level of SOCS3 could be elevated by LXR agonists in HCC cells, Western blot was performed. As shown in Figure 3A and 3B, the LXR agonists GW3965 and TO901317 dose-dependently upregulated SOCS3 protein in HepG2 (Figure 3A) and Hep3B (Figure 3B) cells. As LXR has two isoforms (LXRα and LXRβ), we investigated which isoform is involved in the LXR-mediated induction of SOCS3 using small-interference RNA targeting LXRα and LXRβ respectively. As shown in Figure 3C and 3D, knockdown of LXRα remarkably attenuated GW3965- and TO901317-mediated induction of SOCS3 in HepG2 cells. However, knockdown of LXRβ had no significant influence on GW3965- and TO901317-mediated SOCS3 induction. Collectively, these results indicated that LXR agonists increased the expression of SOCS3 through activating LXRα but not LXRβ.

### Activation of LXR enhances the stability of SOCS3 mRNA

**Figure 4:**
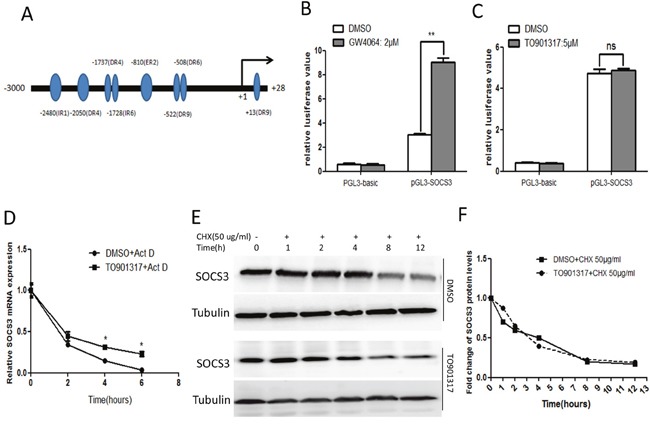
Activation of LXR enhances the stability of SOCS3 mRNA **(A)** The potential LXREs in human *SOCS3* gene promoter region were predicted using bioinformatic analysis (http://www.nubiscan.unibas.ch/). **(B, C)** After transiently transfected with pRL-TK and pGL3-SOCS3 (or the control pGL3-basic) for 12 h, HepG2 cells were treated with 2 μM GW4064 or 5 μM TO901317 (or DMSO) for 18 h. Then the luciferase activity of *SOCS3* gene promoter region was detected by dual luciferase reporter assay system. Data represent the mean ± SD of triplicate experiments. **(D)** After treated with DMSO or TO90137 (5 μM) for 12 h, HepG2 cells were incubated with actinomycin D (Act D, 10 μg/ml) for the indicated times. Then the mRNA level of SOCS3 was examined by qPCR. **(E)** HepG2 cells were treated with TO901317 (5 μM) for 12 h, followed by the incubation with 50 μg/ml cycloheximide (CHX) for the indicated times. Then the level of SOCS3 protein was assayed by Western blot. **(F)** The quantitative analysis of the Western blot results in **(E)** using quantity-one software. The relative value of SOCS3 protein level was normalized by the control tubulin. *P < 0.05; **P < 0.01; ns: no significance.

To explore the underlying mechanism by which LXR induces SOCS3, the potential LXREs in *SOCS3* gene promoter region were predicted using online analysis (http://www.nubiscan.unibas.ch/), and the putative LXREs were shown in Figure 4A. Subsequently, *SOCS3* promoter region (−3000 to +28) was cloned into pGL3-basic vector, and the resulting recombinant plasmid was named pGL3-SOCS3. Previous study has demonstrated that FXR activation enhances the activity of *SOCS3* gene promoter [26], so the activity of *SOCS3* gene promoter in response to LXR agonist was examined with dual luciferase reporter assay, taking the FXR agonist GW4064 as a positive control. As shown in Figure 4B and 4C, GW4064 dramatically increased the activity of *SOCS3* gene promoter, whereas the LXR agonist TO901317 showed no significant influence on the activity of *SOCS3* gene promoter, indicating that LXR upregulated SOCS3 mRNA not via enhancing its transcription. Subsequently, we investigated whether LXR agonist could regulate the stability of SOCS3 mRNA. As shown in Figure 4D, TO901317 markedly increased the mRNA stability of SOCS3. Additionally, the protein stability of SOCS3 was measured using translation inhibitor cycloheximide (CHX). As shown in Figure 4E and 4F, TO901317 could not change the degradation rate of SOCS3, indicating that TO901317 had no significant influence on the protein stability of SOCS3. Taken together, these data indicated that activation of LXR upregulated SOCS3 through enhancing its mRNA stability.

### LXR activation decreases cyclin D1 and increases p21 and p27 via inducing SOCS3

**Figure 5:**
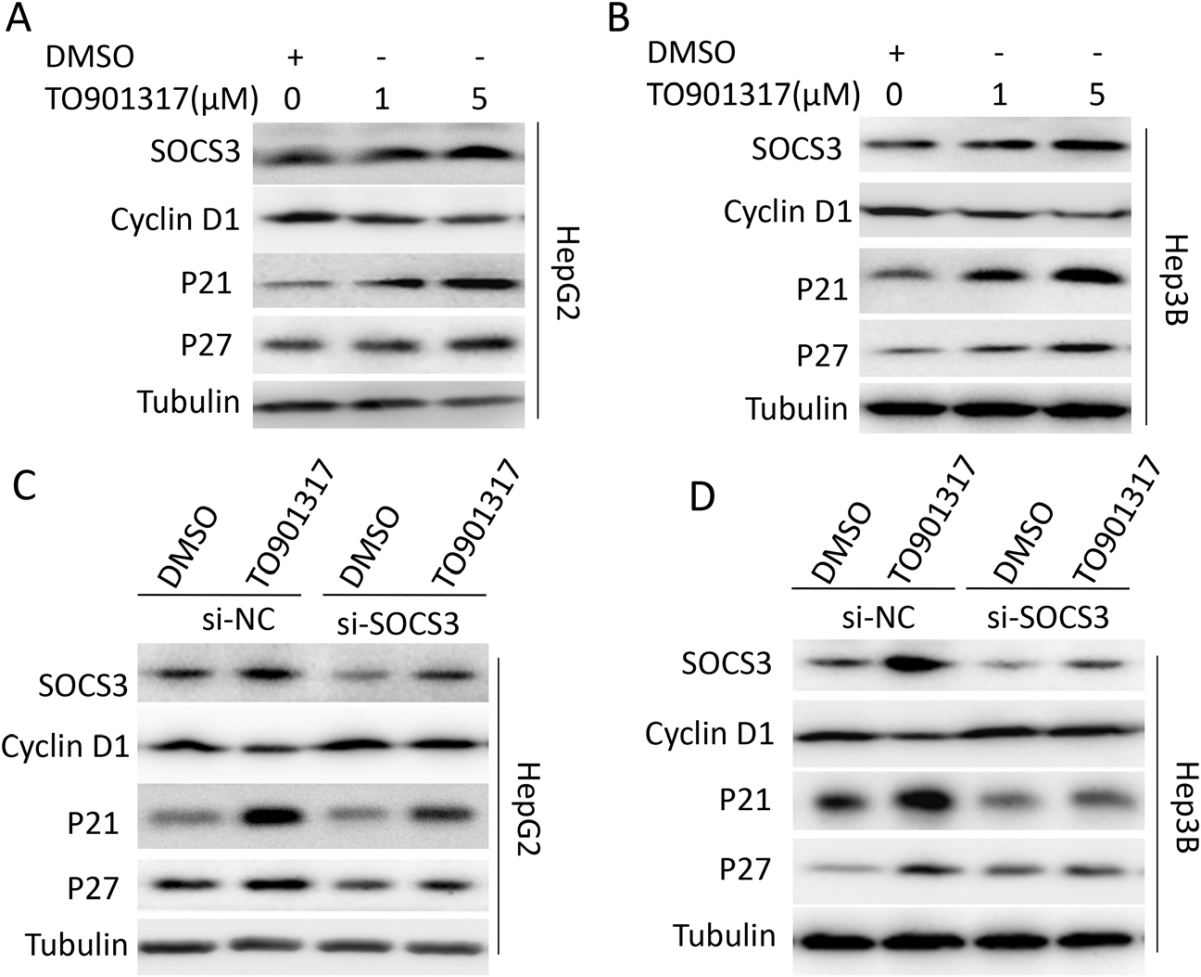
LXR activation decreases cyclin D1 and increases p21 and p27 via inducing SOCS3 **(A, B)** After cultured in complete medium for 12 h, HepG2 **(A)** and Hep3B **(B)** cells were treated with DMSO or different concentrations (1 μM and 5μM) of LXR agonist TO901317 for 24 h. Then the level of SOCS3 protein was measured by Western blot. **(C, D)** After transiently transfected with SOCS3 siRNA (si-SOCS3) or negative control siRNA (si-NC) for 12 h, HepG2 **(C)** and Hep3B **(D)** cells were treated with DMSO or TO901317 (5 μM) for 24 h. Then the protein levels of SOCS3, cyclinD1, p21 and p27 were assayed by Western blot.

Previous studies have reported that activation of LXR inhibits the proliferation of cancer cells through repressing cyclin D1 and increasing p21 and p27 [11, 27]. Therefore, the levels of cyclin D1, p21 and p27 in HCC cells were measured after treatment with LXR agonist. As shown in Figure 5A and 5B, activation of LXR by TO901317 significantly decreased cyclin D1, while increased p21 and p27 in HepG2 (Figure 5A) and Hep3B (Figure 5B) cells, which was dramatically attenuated by knockdown of SOCS3 (Figure 5C and 5D), indicating that LXR upregulated cyclin D1 while downregulated p21 and p27 via inducing SOCS3.

### Knockdown of SOCS3 attenuates LXR-induced G1/S cell cycle arrest and anti-proliferation effects

**Figure 6:**
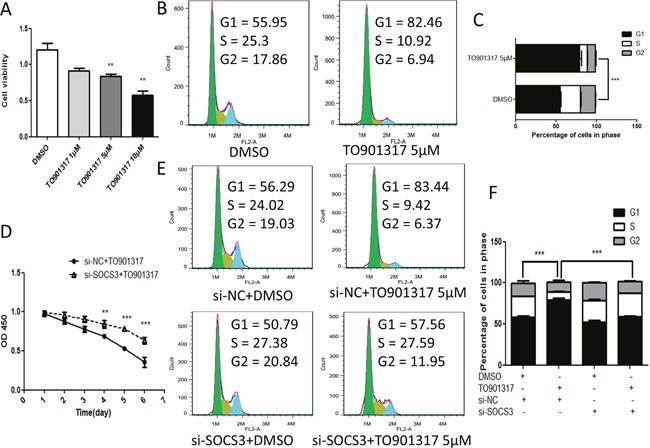
Knockdown of SOCS3 attenuates LXR-induced G1/S cell cycle arrest and anti-proliferation effects **(A)** After cultured in 96-well plates for 12 h, HepG2 cells were treated with DMSO or different concentrations of TO901317 for 48 h. Then the cell viability was determined by CCK-8 assay. **(B, C)** After cultured in 6-well plates for 12 h, HepG2 cells were treated with DMSO or TO901317 (5 μM) for 48 h. Then the cells were stained with PI, and the cell cycle was analyzed using flow cytometry **(B)**. The percentage of cells in each cell cycle phase was quantifed **(C)**. **(D)** After grown to 80% confluence in 96-well plates, HepG2 cells were transiently transfected with SOCS3 siRNA (si-SOCS3) or negative control siRNA (si-NC) for 12 h. Then the cells were treated with DMSO or TO901317 (5 μM) for the indicated times. Subsequently, the cell viability was detected using CCK-8 assay. **(E, F)** After grown to 80% confluence in 6-well plates, HepG2 cells were transiently transfected with si-SOCS3 or si-NC for 12 h, and then treated with DMSO or TO901317 (5 μM) for 48 h. Next, the cells were stained with PI, and the cell cycle was analyzed using flow cytometry **(E)**. The percentage of cells in each cell cycle phase was quantifed **(F)**. **P < 0.01; ***P < 0.001.

It has been reported that LXR agonists suppress the proliferation of HCC cells [11], so the cell viability and cell cycle of HCC cells were measured by CCK8 and flow cytometry respectively after treatment with TO901317. As shown in Figure 6A, activation of LXR by TO901317 dose-dependently inhibited the growth of HepG2 cells, which was markedly attenuated by knockdown of SOCS3 (Figure 6D). Moreover, flow cytometry showed that TO901317 led to the cell cycle arrest at G1/S phase (Figure 6B and 6C), which was dramatically alleviated by knockdown of SOCS3 (Figure 6E and 6F). Collectively, these results indicated that LXR induced G1/S cell cycle arrest and repressed HCC cell growth via elevating SOCS3.

### LXR agonist suppresses the growth of HCC xenografts and enhances SOCS3 expression *in vivo*

**Figure 7:**
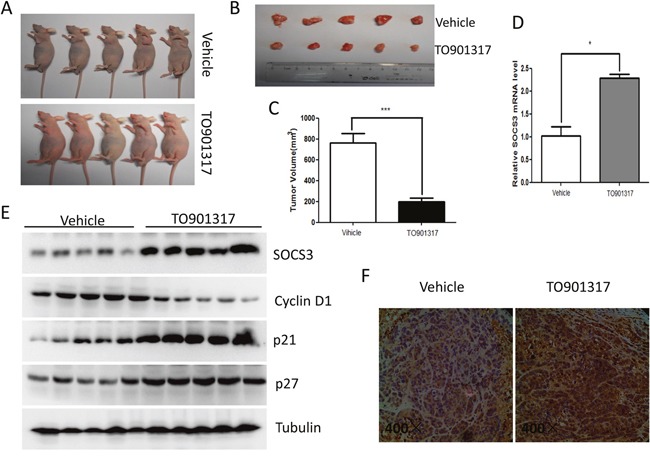
LXR agonist suppresses the growth of HCC xenografts and enhances SOCS3 expression *in vivo* 1×10^6^ HepG2 cells were implanted into the left axilla of each 4-weeks old male nude mouse. When palpable tumors were formed, TO901317 (25 mg/kg/d) or vehicle (soyabean oil) was injected into enterocoelia of the nude mice every day for 2 weeks (n=5 per group). Then the mice were sacrificed **(A)** and the xenograft tumors were excised and photographed **(B)**. Subsequently, the xenograft tumor size was measured and analyzed **(C)**. The level of SOCS3 mRNA in the HCC xenografts was determined by qPCR **(D)**, and the protein levels of SOCS3, cyclin D1, p21 and p27 were detected by Western blot **(E)**. **(F)** Immunohistochemistry analysis of SOCS3 protein in the HCC xenografts. *P < 0.05; ***P < 0.001.

As shown in Figure 7A-7C, treatment with LXR agonist TO901317 markedly inhibited the growth of HCC xenografts in nude mice. qPCR (Figure 7D), Western blot (Figure 7E) and IHC (Figure 7F) revealed that the mRNA and protein levels of SOCS3 were notably upregulated by TO901317 in the HCC xenografts. Moreover, TO901317 dramatically decreased cylcin D1, while significantly increased p21 and p27 in the HCC xenografts (Figure 7E). Collectively, these results indicated that activation of LXR induced SOCS3 and repressed the growth of HCC *in vivo*.

## DISCUSSION

HCC is the second leading cause of cancer-related death in the world [28, 29]. Many tumor suppressor genes are silenced during the development and progression of HCC, and reactivation/restoration of these cancer suppressor genes has shown a promising therapeutic strategy for HCC prevention and treatment [30–32]. In this study, we found that SOCS3 (a tumor suppressor molecule) was significantly downregulated in HCC. Re-expression of SOCS3 by LXR agonists led to cell cycle arrest through increasing p21 and p27 and decreasing cyclin D1, which resulted in the growth inhibition of HCC cells.

SOCS3, as a tumor suppressor, has been reported to be silenced in several human cancers including HCC [33, 34]. Previous studies have demonstrated that the methylation of *SOCS3* gene promoter region leads to its downregulation in HCC, and re-expression of SOCS3 results in apoptosis and cell cycle arrest [35–37]. In this study, we showed that the expression of SOCS3 in HCC tissues was dramatically decreased, which was in line with the previous report [26]. Moreover, we observed that the level of SOCS3 was also downregulated in five HCC cell lines compared to the relatively normal cell line L02. Interestingly, the level of SOCS3 in Huh7 cells was relatively higher compared to other HCC cell lines. The reason for the high level of SOCS3 in Huh7 cells may be attributed to the various methylation status of *SOCS3* gene promoter region in different HCC cell lines [35]. Among the examined five HCC cell lines, HepG2 and Hep3B cells showed the lowest level of SOCS3, so we chose these two cell lines to investigate the LXR-mediated upregulation of SOCS3 in this study. It has been reported that several nuclear receptors including FXR [26], ERα [38], PPARβ/γ [39, 40], participates in regulating SOCS3. However, it is still unknown whether the nuclear receptor LXR can exert its anti-HCC effects via elevating SOCS3 even though previous studies have shown that LXR suppresses the growth of HCC cells via regulating its downstream target genes such as FOXM1 [11] and ABCA1 [41]. In this study, we demonstrated that upregulation of SOCS3 is a novel mechanism by which LXR inhibits the proliferation of HCC cells. Furthermore, we proved that the cell cycle related proteins (cyclin D1, p21 and p27) are influenced by the LXR-induced SOCS3. Nevertheless, the detailed mechanism(s) by which SOCS3 regulates the levels of cyclin D1, p21 and p27 need(s) to be further studied.

In the present study, we for the first time revealed that LXR activation elevated the level of SOCS3 in HCC cells. Mechanistically, activation of LXR enhanced the mRNA stability of SOCS3 but had no significant influence on the transcriptional activity of *SOCS3* gene promoter. However, it should be noted that we only cloned *SOCS3* promoter region harboring the sequence −3000 to +28, so it remains to be further investigated whether LXR could regulate the transcriptional activity of the longer *SOCS3* promoter region beyond −3000 to +28. Interestingly, we found that activation of LXR could stabilize the SOCS3 mRNA. A previous report has shown that MEK1 and Erk1/2 can mediate the stabilization of SOCS3 transcript in primary human and murine hepatocytes [42]. In addition, TNF-alpha has been reported to enhance the mRNA stability of SOCS3 via MKK6/p38/MK2 cascade [43]. However, whether MEK1-Erk1/2 and MKK6/p38/MK2 are involved in the LXR-mediated stability of SOCS3 mRNA needs to be further explored.

LXRα and LXRβ, two isoforms of LXR, have been reported to repress the proliferation of cancer cells [44]. However, in this study, only LXRα was demonstrated to affect the level of SOCS3, indicating that the LXRα-mediated induction of SOCS3 is responsible for the anti-HCC effects of LXR agonists even though LXRβ has the function of anti-cancer [45]. As LXR participates in the reverse cholesterol transport [46, 47], LXR agonists have received accumulating attention for their benefits in correcting metabolic disorders characterized by cholesterol accumulation. Recently, the anti-cancer property of LXR becomes a hot area because mounting studies have proved that LXR agonists hold the new function of anti-proliferation in various cancer cells [48]. However, it needs to be noted that LXR agonists have some side-effects such as promoting hepatic lipogenesis [49, 50]. Therefore, it is urgent to identify and develop novel LXR ligands specifically for cancer therapy to minimize or bypass any potential side effects including hepatotoxicity. In summary, this study indicates that “LXRα-SOCS3-cyclin D1/p21/p27” is a novel pathway by which LXR exerts its anti-HCC effects, suggesting that the pathway may be a new potential therapeutic target for HCC treatment/prevention.

## MATERIALS AND METHODS

### Materials

GW3965, TO901317, GW4064 and dimethylsulphoxide (DMSO) were purchased from Sigma-Aldrich (St Louis, MO, USA). Actinomycin D (Act D) and cycloheximide (CHX) were from Xiya Reagent (Chengdu, china). Lipofectamin 2000, Optim-medium, M-MLV, Oligo dT, dNTPs and DTT were from Invitrogen (Carlsbad, CA, USA). RNA Out was from TIANDZ (Beijing, China). SYBR Green Mix, restriction endonucleases *Kpn* I and *Nhe* I, and PrimeSTAR HS DNA polymerase were from Takara (Kyoto, Japan). RIPA lysis buffer and BCA protein assay kit were bought from Beyotime Biotechnology (Shanghai, China). The protease inhibitor cocktail was from Roche (Basel, Switzerland). Cell Counting Kit-8 (CCK-8) was from Dojindo (Kumamoto, Japan). PGL3-basic vector, pRL-TK vector and Dual-Luciferase Reporter Assay System were from Promega (Madison, WI, USA). SiRNAs for LXRα, LXRβ, SOCS3 and control siRNA were synthesized by GenePharma (Shanghai, China).

### HCC tissues and adjacent noncancerous specimens

A total of 30 HCC tissues and the corresponding adjacent noncancerous specimens were obtained from Department of Hepatobiliary Surgery, Xinqiao Hospital, Third Military Medical University (Chongqing, China). Fresh indentified HCC tissue samples were collected and embedded by paraffin. The protocols for handling the HCC specimens were approved by the IBC (Institutional Biosafety Committee) and ethical committees of the Third Military Medical University. Written informed consent was provided to every enrolled patient.

### Cell culture

Human HCC cell lines (HepG2, Hep3B, PLC, Huh7, smmc7721) and the relatively normal hepatic cell line L02 were originally tested and authenticated by American Type Culture Collection (Manassas, VA, USA) and passaged less than 6 months in the lab. Briefly, when the cells were grown to 80% confluence in 75 cm^2^ flask, the culture medium was removed and discarded, and then the cells were washed with PBS for two times. Thereafter, 2 ml 0.25% trypsin was added to the flask to disperse the cell layer. After that, 4 ml complete medium was added and the cells were aspirated by gently pipetting. Lastly, appropriate aliquots of the cell suspension were transferred to new flasks to cultivate. All the cells were cultured in DMEM medium containing 10% fetal bovine serum (FBS) at 37° in 5% CO_2_ incubator.

### Immunohistochemistry (IHC)

The specimens were harvested and then fixed in 4% paraformaldehyde overnight. Subsequently, the tissues were embedded in paraffin, and then cut at 5 μm thickness for histological analysis. Tumor sections were immunostained with Histostain-Plus Kits from Beijing Zhongshan Biotechnologies (SP-9000) according to the manufacture's instructions. Briefly, sections were treated with 3% H_2_O_2_ to block endogenous peroxidase, followed by the incubation with 0.1% trypsin for antigen demasking. Then the sections were blocked using 5% goat serum (Beijing Zhongshan Biotechnologies, SP-9000) for 30 min at 37°C, followed by the incubation with the indicated primary antibodies overnight at 4°C. After warming and cleaning, the sections were incubated with respective secondary antibodies for 30 min at 37°C. Lastly, the sections were counterstained with Alcian blue.

### quantitative PCR (qPCR)

Total RNA was extracted using RNA OUT reagent according to the manufacturer's instructions. After that, the first-strand cDNA was synthesized using reverse transcriptase M-MLV. qPCR was performed using 2×SYBR qPCR mix (Takara, Kyoto, Japan). Each amplification mixture (25 μl) contains 25 ng of cDNA, 1 μl of 10 μM primers and 12.5 μl of SYBR qPCR mix. Amplification is performed using the following conditions: 95°C for 5 min, and 40 cycles of 95°C for 15 s and 60°C for 1 min. The corresponding primer sets were listed as follows.

**Table:** 

Gene	Forward primer	Reversed primer
*SOCS3*	5′-ATCCTGGTGACATGCTCCTC-3′	5′-GGCACCAGGTAGACTTTGGA-3′
*β-actin*	5′-GTGAAGGTGACAGCAGTCGGTT-3′	5′-GAAGTGGGGTGGTTTTAGGA-3′
*ABCA1*	5′-GAGAGGAGTCCCAGAGAAAGAAG-3′	5′-ACTACTGATCTCCCCTCCTTGAC-3′

### Western blot

Cells or tissues were lysed, and then the protein concentrations were detected by BCA kit. After transferred to polyvinylidene difluoride (PVDF) membranes, the target proteins were separately detected by the primary antibodies including rabbit anti-SOCS3 (ab16030), mouse anti-LXRα (ab41902), rabbit anti-LXRβ (ab117881), rabbit ant-cyclinD1 (ab16663), rabbit anti-p21 (SC397), rabbit anti-p27 (CST2552), and mouse anti-Tubulin (AT819). The HRP labeled goat anti-rabbit and goat anti-mouse Ig G were used as secondary antibodies.

### Si-RNA assay

HepG2 and Hep3B cells were separately cultured in 6-well plates overnight, and then the corresponding siRNA or control siRNA (si-NC) was transfected into the cells by lipofectamin 2000 according to the manufacturer's instructions. After 6 h, the transfection medium was removed and cultured in DMEM with 10% FBS for another 6 h. Subsequently, the cells were treated with LXR agonists or vehicle control for 24 h, and then the corresponding assays were performed. The sequences of siRNAs for targeted genes were listed as follows.

**Table:** 

target	siRNA sequence
LXRα	5′-GAAGAACAGAUCCGCUUGATT-3′
LXRβ	5′-UGAGGAGCAGAUUCGGAAGTT-3′
SOCS3	5′-CACCUGGACUCCUAUGAGATT-3′
NC	5′-UUCUCCGAACGUGUCACGUTT-3′

### Plasmid construction

HepG2 genome DNA was extracted using genome DNA extracting kit (TianGen, Beijing), then human *SOCS3* promoter region (−3000 to +28) was amplified by PCR with the oligonucleotides 5′-CGGGGTACCACAAATAAGGAAACCGAGGCA-3′ (forward primer) and 5′-CTAGCTAGCTAGCGGAGCAGGGAGTCCAAGT-3′ (reverse primer) using genomic DNA of HepG2 cells. After digested with *Kpn* I and *Nhe* I, the target amplified fragment was cloned into pGL3-basic vector, and the resulting recombinant plasmid was named as pGL3-SOCS3. The details were supplied in Supplementary Figure 1.

### Dual luciferase reporter assay

After grown to 80% confluence in 96-well plates, HepG2 and Hep3B cells were transfected with pGL3-SOCS3 and pRL-TK plasmids using lipofectamin 2000 according to the manufacturer's instructions. Six hours later, the transfection medium was replaced by DMEM with 10% FBS and cultured for another 6 h. Then the cells were treated with 5μM LXR agonist or vehicle control DMSO for 18 h. Subsequently, the luciferase activity was determined and normalized according to the protocol of dual luciferase reporter assay system. Briefly, 50 μl/well Dual-Glo^®^ Luciferase Assay Reagent was added to the 96-well plate and incubated at room temperature for 10 minutes, then the firefly luminescence was measured with SpectraMax L Microplate Reader (Molecular Devices, Sunnyvale, CA, USA) at 570 nm. After that, 50 μl/well Dual-Glo^®^ Stop & Glo^®^ Reagent was add to the plate and incubated at room temperature for 10 minutes, then the renilla luminescence was detected with the above Microplate Reader at 470 nm. The ratio of firefly/renilla luminescence for each well was calculated, and then the test sample ratio was normalized against the control ratio. Each experiment was performed in triplicate.

### CCK-8 assay

HepG2 cells were cultured in 96-well plates overnight and then treated with vehicle control DMSO or various concentrations of LXR agonist TO901317 for 48 h. Subsequently, 10 μl/well of CCK-8 solution was added to the plate and incubated for 30 min at 37°C. The value of formazan dye generated by cellular dehydrogenase activity was measured for absorbance at 450 nm (OD450) by a microplate reader (Molecular Devices, Sunnyvale, CA, USA). The OD450 value of each well represented the survival of HCC cells. The OD450 value of test well was normalized with that of control well. Each experiment was done in triplicate.

### Flow cytometry assay

The HCC cells were trypsinized and collected into centrifuge tubes. Subsequently, the cells were washed with phosphate-buffered saline (PBS) for 2 times, and then resuspended in 75% ethanol and fixed overnight at 4°C. Next, the cells were incubated with RNaseA (50 μg/ml) (Sigma, St Louis, MO, USA) in PBS at 37°C for 30 min. Thereafter, the cells were incubated with propidium iodide (PI, 50 μg/ml) at darkness for 10 min, and then the cell cycle was assayed by Flow cytometer (Becton, Dickinson and Company, Franklin Lakes, NJ, USA).

### Animal experiment

Four-week-old male nude mice were obtained from Beijing Huafukang Bioscience (Beijing, China), and housed and cared for under the regulations of the guidelines of the Animal Care and Ethics Committee of Third Military Medical University (Chongqing, China). One week later, 1×10^6^ HepG2 cells were subcutaneously injected into the right axilla of each nude mouse. When palpable tumors formed, the mice were randomly divided into two groups. One group was intraperitonealy injected soyabean oil and the other group was intraperitonealy injected TO901317 (25 mg/kg) for 2 weeks. After that, the mice were sacrificed and the xenograft tumors were harvested. The tumor volume was calculated using the formula of volume=width^2^×length×1/2. qPCR, Western blot and IHC were conducted to determine the mRNA and protein levels of the target molecule in the xenografts.

### Statistic analysis

The data were expressed as mean ± SD. One-way ANOVA and *t*-test were used to analyze the variance. *P* < 0.05 was defined as statistically significant.

## SUPPLEMENTARY MATERIALS FIGURES AND TABLES



## References

[1] Lin CY, Gustafsson JA (2015). Targeting liver X receptors in cancer therapeutics. Nat Rev Cancer.

[2] Calkin AC, Tontonoz P (2012). Transcriptional integration of metabolism by the nuclear sterol-activated receptors LXR and FXR. Nat Rev Mol Cell Biol.

[3] Repa JJ, Mangelsdorf DJ (2000). The role of orphan nuclear receptors in the regulation of cholesterol homeostasis. Annu Rev Cell Dev Biol.

[4] Ducheix S, Montagner A, Theodorou V, Ferrier L, Guillou H (2013). The liver X receptor: a master regulator of the gut-liver axis and a target for non alcoholic fatty liver disease. Biochem Pharmacol.

[5] Ma Z, Deng C, Hu W, Zhou J, Fan C, Di S, Liu D, Yang Y, Wang D. (2017). Liver X Receptors and their agonists: targeting for cholesterol homeostasis and cardiovascular diseases. Curr Issues Mol Biol.

[6] Fukuchi J, Kokontis JM, Hiipakka RA, Chuu CP, Liao S (2004). Antiproliferative effect of liver X receptor agonists on LNCaP human prostate cancer cells. Cancer Res.

[7] Vedin LL, Lewandowski SA, Parini P, Gustafsson JA, Steffensen KR (2009). The oxysterol receptor LXR inhibits proliferation of human breast cancer cells. Carcinogenesis.

[8] Scoles DR, Xu X, Wang H, Tran H, Taylor-Harding B, Li A, Karlan BY (2010). Liver X receptor agonist inhibits proliferation of ovarian carcinoma cells stimulated by oxidized low density lipoprotein. Gynecol Oncol.

[9] Pencheva N, Buss CG, Posada J, Merghoub T, Tavazoie SF (2014). Broad-spectrum therapeutic suppression of metastatic melanoma through nuclear hormone receptor activation. Cell.

[10] Lo Sasso G, Bovenga F, Murzilli S, Salvatore L, Di Tullio G, Martelli N, D'Orazio A, Rainaldi S, Vacca M, Mangia A, Palasciano G, Moschetta A (2013). Liver X receptors inhibit proliferation of human colorectal cancer cells and growth of intestinal tumors in mice. Gastroenterology.

[11] Hu C, Liu D, Zhang Y, Lou G, Huang G, Chen B, Shen X, Gao M, Gong W, Zhou P, Dai S, Zeng Y, He F (2014). LXRalpha-mediated downregulation of FOXM1 suppresses the proliferation of hepatocellular carcinoma cells. Oncogene.

[12] Yasukawa H, Sasaki A, Yoshimura A (2000). Negative regulation of cytokine signaling pathways. Annu Rev Immunol.

[13] Yasukawa H, Misawa H, Sakamoto H, Masuhara M, Sasaki A, Wakioka T, Ohtsuka S, Imaizumi T, Matsuda T, Ihle JN, Yoshimura A (1999). The JAK-binding protein JAB inhibits Janus tyrosine kinase activity through binding in the activation loop. EMBO J.

[14] Ram PA, Waxman DJ (1999). SOCS/CIS protein inhibition of growth hormone-stimulated STAT5 signaling by multiple mechanisms. J Biol Chem.

[15] Linossi EM, Nicholson SE (2012). The SOCS box-adapting proteins for ubiquitination and proteasomal degradation. IUBMB Life.

[16] Carow B, Rottenberg ME (2014). SOCS3, a major regulator of infection and inflammation. Front Immunol.

[17] Baker BJ, Akhtar LN, Benveniste EN (2009). SOCS1 and SOCS3 in the control of CNS immunity. Trends Immunol.

[18] Galic S, Sachithanandan N, Kay TW, Steinberg GR (2014). Suppressor of cytokine signalling (SOCS) proteins as guardians of inflammatory responses critical for regulating insulin sensitivity. Biochem J.

[19] Stahl A, Joyal JS, Chen J, Sapieha P, Juan AM, Hatton CJ, Pei DT, Hurst CG, Seaward MR, Krah NM, Dennison RJ, Greene ER, Boscolo E (2012). SOCS3 is an endogenous inhibitor of pathologic angiogenesis. Blood.

[20] Gao Z, Jin YQ, Wu W (2017). SOCS3 treatment prevents the development of alopecia areata by inhibiting CD8+ T cell-mediated autoimmune destruction. Oncotarget.

[21] Lesina M, Kurkowski MU, Ludes K, Rose-John S, Treiber M, Kloppel G, Yoshimura A, Reindl W, Sipos B, Akira S, Schmid RM, Algul H (2011). Stat3/Socs3 activation by IL-6 transsignaling promotes progression of pancreatic intraepithelial neoplasia and development of pancreatic cancer. Cancer Cell.

[22] Barclay JL, Anderson ST, Waters MJ, Curlewis JD (2009). SOCS3 as a tumor suppressor in breast cancer cells, and its regulation by PRL. Int J Cancer.

[23] Sutherland KD, Lindeman GJ, Choong DY, Wittlin S, Brentzell L, Phillips W, Campbell IG, Visvader JE (2004). Differential hypermethylation of SOCS genes in ovarian and breast carcinomas. Oncogene.

[24] Ogata H, Kobayashi T, Chinen T, Takaki H, Sanada T, Minoda Y, Koga K, Takaesu G, Maehara Y, Iida M, Yoshimura A (2006). Deletion of the SOCS3 gene in liver parenchymal cells promotes hepatitis-induced hepatocarcinogenesis. Gastroenterology.

[25] Chawla A, Boisvert WA, Lee CH, Laffitte BA, Barak Y, Joseph SB, Liao D, Nagy L, Edwards PA, Curtiss LK, Evans RM, Tontonoz P (2001). A PPAR gamma-LXR-ABCA1 pathway in macrophages is involved in cholesterol efflux and atherogenesis. Mol Cell.

[26] Guo F, Xu Z, Zhang Y, Jiang P, Huang G, Chen S, Lyu X, Zheng P, Zhao X, Zeng Y, Wang S, He F (2015). FXR induces SOCS3 and suppresses hepatocellular carcinoma. Oncotarget.

[27] Rough JJ, Monroy MA, Yerrum S, Daly JM (2010). Anti-proliferative effect of LXR agonist T0901317 in ovarian carcinoma cells. J Ovarian Res.

[28] El-Serag HB (2012). Epidemiology of viral hepatitis and hepatocellular carcinoma. Gastroenterology.

[29] Ferlay J, Soerjomataram I, Dikshit R, Eser S, Mathers C, Rebelo M, Parkin DM, Forman D, Bray F (2015). Cancer incidence and mortality worldwide: sources, methods and major patterns in GLOBOCAN 2012. Int J Cancer.

[30] Ranzani M, Cesana D, Bartholomae CC, Sanvito F, Pala M, Benedicenti F, Gallina P, Sergi LS, Merella S, Bulfone A, Doglioni C, von Kalle C, Kim YJ (2013). Lentiviral vector-based insertional mutagenesis identifies genes associated with liver cancer. Nat Methods.

[31] Niu ZS, Niu XJ, Wang WH (2016). Genetic alterations in hepatocellular carcinoma: an update. World J Gastroenterol.

[32] Schulze K, Nault JC, Villanueva A (2016). Genetic profiling of hepatocellular carcinoma using next-generation sequencing. J Hepatol.

[33] Ogata H, Chinen T, Yoshida T, Kinjyo I, Takaesu G, Shiraishi H, Iida M, Kobayashi T, Yoshimura A (2006). Loss of SOCS3 in the liver promotes fibrosis by enhancing STAT3-mediated TGF-beta1 production. Oncogene.

[34] Tischoff I, Hengge UR, Vieth M, Ell C, Stolte M, Weber A, Schmidt WE, Tannapfel A (2007). Methylation of SOCS-3 and SOCS-1 in the carcinogenesis of Barrett's adenocarcinoma. Gut.

[35] Niwa Y, Kanda H, Shikauchi Y, Saiura A, Matsubara K, Kitagawa T, Yamamoto J, Kubo T, Yoshikawa H (2005). Methylation silencing of SOCS-3 promotes cell growth and migration by enhancing JAK/STAT and FAK signalings in human hepatocellular carcinoma. Oncogene.

[36] Jiang BG, Wang N, Huang J, Yang Y, Sun LL, Pan ZY, Zhou WP (2017). Tumor SOCS3 methylation status predicts the treatment response to TACE and prognosis in HCC patients. Oncotarget.

[37] Yuan K, Lei Y, Chen HN, Chen Y, Zhang T, Li K, Xie N, Wang K, Feng X, Pu Q, Yang W, Wu M, Xiang R, Nice EC, Wei Y, Huang C (2016). HBV-induced ROS accumulation promotes hepatocarcinogenesis through Snail-mediated epigenetic silencing of SOCS3. Cell Death Differ.

[38] Matthews J, Almlof T, Kietz S, Leers J, Gustafsson JA (2005). Estrogen receptor-alpha regulates SOCS-3 expression in human breast cancer cells. Biochem Biophys Res Commun.

[39] Serrano-Marco L, Rodriguez-Calvo R, El Kochairi I, Palomer X, Michalik L, Wahli W, Vazquez-Carrera M (2011). Activation of peroxisome proliferator-activated receptor-beta/-delta (PPAR-beta/-delta) ameliorates insulin signaling and reduces SOCS3 levels by inhibiting STAT3 in interleukin-6-stimulated adipocytes. Diabetes.

[40] Berger H, Vegran F, Chikh M, Gilardi F, Ladoire S, Bugaut H, Mignot G, Chalmin F, Bruchard M, Derangere V, Chevriaux A, Rebe C, Ryffel B (2013). SOCS3 transactivation by PPARgamma prevents IL-17-driven cancer growth. Cancer Res.

[41] Kaneko T, Kanno C, Ichikawa-Tomikawa N, Kashiwagi K, Yaginuma N, Ohkoshi C, Tanaka M, Sugino T, Imura T, Hasegawa H, Chiba H (2015). Liver X receptor reduces proliferation of human oral cancer cells by promoting cholesterol efflux via up-regulation of ABCA1 expression. Oncotarget.

[42] Ehlting C, Bohmer O, Hahnel MJ, Thomas M, Zanger UM, Gaestel M, Knoefel WT, Schulte Am Esch J, Haussinger D, Bode JG (2015). Oncostatin M regulates SOCS3 mRNA stability via the MEK-ERK1/2-pathway independent of p38(MAPK)/MK2. Cell Signal.

[43] Ehlting C, Lai WS, Schaper F, Brenndorfer ED, Matthes RJ, Heinrich PC, Ludwig S, Blackshear PJ, Gaestel M, Haussinger D, Bode JG (2007). Regulation of suppressor of cytokine signaling 3 (SOCS3) mRNA stability by TNF-alpha involves activation of the MKK6/p38MAPK/MK2 cascade. J Immunol.

[44] Vedin LL, Gustafsson JA, Steffensen KR (2013). The oxysterol receptors LXRalpha and LXRbeta suppress proliferation in the colon. Mol Carcinog.

[45] Courtaut F, Derangere V, Chevriaux A, Ladoire S, Cotte AK, Arnould L, Boidot R, Rialland M, Ghiringhelli F, Rebe C (2015). Liver X receptor ligand cytotoxicity in colon cancer cells and not in normal colon epithelial cells depends on LXRbeta subcellular localization. Oncotarget.

[46] Ito A, Hong C, Oka K, Salazar JV, Diehl C, Witztum JL, Diaz M, Castrillo A, Bensinger SJ, Chan L, Tontonoz P (2016). Cholesterol accumulation in CD11c+ immune cells is a causal and targetable factor in autoimmune disease. Immunity.

[47] Lo Sasso G, Murzilli S, Salvatore L, D'Errico I, Petruzzelli M, Conca P, Jiang ZY, Calabresi L, Parini P, Moschetta A (2010). Intestinal specific LXR activation stimulates reverse cholesterol transport and protects from atherosclerosis. Cell Metab.

[48] Lin CY, Vedin LL, Steffensen KR (2016). The emerging roles of liver X receptors and their ligands in cancer. Expert Opin Ther Targets.

[49] Beaven SW, Matveyenko A, Wroblewski K, Chao L, Wilpitz D, Hsu TW, Lentz J, Drew B, Hevener AL, Tontonoz P (2013). Reciprocal regulation of hepatic and adipose lipogenesis by liver X receptors in obesity and insulin resistance. Cell Metab.

[50] Wu J, Wang C, Li S, Wang W, Li J, Chi Y, Yang H, Kong X, Zhou Y, Dong C, Wang F, Xu G, Yang J (2013). Thyroid hormone-responsive SPOT 14 homolog promotes hepatic lipogenesis, and its expression is regulated by liver X receptor alpha through a sterol regulatory element-binding protein 1c-dependent mechanism in mice. Hepatology.

