# Supplementary material for: Induction of SOCS3 by liver X receptor suppresses the proliferation of hepatocellular carcinoma cells
Source: Oncotarget. 2017 Jul 18;8(38):64083–94. doi: 10.18632/oncotarget.19321 (PMC5609985; doi:10.18632/oncotarget.19321)
Supplement: Supplementary file 1 [file oncotarget-08-64083-s001.pdf]

Induction of SOCS3 by liver X receptor suppresses the proliferation of hepatocellular carcinoma cells

SUPPLEMENTARY FIGURE

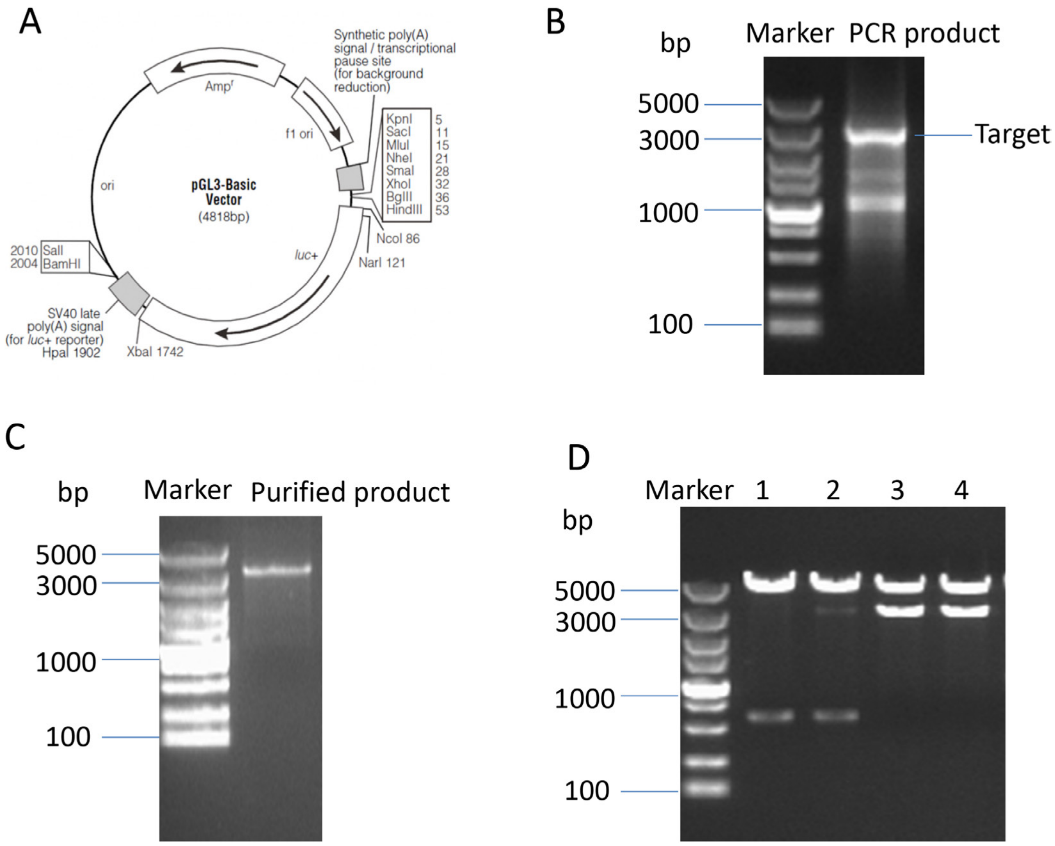

Supplementary Figure 1: Construction of the recombinant plasmid containing SOCS3 promoter region. (A) The map of the pGL3-Basic vector. **(B)** Human SOCS3 promoter region (-3000 to +28) was amplified by PCR using the genomic DNA derived from HepG2 cells as template. Then the PCR products were fractionated with a 1% agarose gel and the bands were visualized with ethidium bromide (EB). The target band at about 3000 bp was sectioned and purified. Subsequently, the purified target fragment was identified by 1% agarose gel **(C)**. **(D)** After digested with *Kpn* I and *Nhe* I, the target fragment was ligated into pGL3-basic vector using T4 DNA ligase. After transformed into *E. coli* DH5-α, the recombinants were selected. Then the recombinant plasmids were extracted and identified with *Kpn* I and *Nhe* I (3 and 4 represented the positive recombinant plasmids).
